# Annual Address of the President of Fulton County Medical Society

**Published:** 1909-01

**Authors:** A. W. Stirling

**Affiliations:** M. D., C. M., Edin; D. P. H., Eng; Atlanta, Ga.


### Journal-Record of Medicine

Successor to Atlanta Medical and Surgical Journal, Established 1855, and Southern Medical Record, Established 1870.

**OWNED BY THE ATLANTA MEDICAL JOURNAL CO.**

**Published Monthly.**

*Official Organ Fulton County Medical Society, State Examining Board, Presbyterian Hospital, Etc.*

EDGAR G. BALLENGER, M. D., Editor.
BERNARD WOLFF, M. D., Supervising Editor.
A. W. STIRLING, M. D., C. M., D. P. H., J. S. HURT, B. Ph., M. D, Associate Editors.
E. W. ALLEN, Business Manager.

COLLABORATORS

DR. W. F. WESTMORLAND, General Surgery.
F. W. McRAE, M. D., Abdominal Surgery.
H. F. HARRIS, M. D., Pathology and Bacteriology.
E. B. BLOCK, M. D., Diseases of the Nervous System.
MICHEL HOKE, M. D., Orthopedic Surgery.
CYRUS W. STRICKLER, M. D., Legal Medicine and Medical Legislation.
E. C. DAVIS, A. B., M. D., Obstetrics.
E. G. JONES, A. B., M. D., Gynecology.
R. T. DORSEY, Jr., B. S. M. D., Medicine.
L. M. GAINES, A. B., M. D., Internal Medicine.
J. N. LeCONTE, M. D., Diseases of the Stomach and Intestines
L. B. CLARKE, M. D., Pediatrics.
EDGAR PAULIN, M. D., Opsonic Medicine.
R. R. DALY, M. D., Medical Society.
A. W. STIRLING, M. D., Etc., Diseases of the Eye, Ear, Nose and Throat.
BERNARD WOLFF, M. D., Diseases of the Skin.
E. G. BALLENGER, M. D., Diseases of the Genito-Urinary Organs.

VOL. XI. JANUARY, 1909. No. 10

### ANNUAL ADDRESS OF THE PRESIDENT OF FULTON COUNTY MEDICAL SOCIETY.

BY A. W STIRLING, M. D., C. M., EDIN; D. P. H., ENG; ATLANTA, GA.

Gentlemen: The president of our society is admonished in chapter IV, section 2, of the By-Laws in the following words: "He shall, at the close of his administration, at the regular meeting for the election of officers, submit to the society an annual message, devoted to an account of his stewardship, and to the discussion of the interests, objects, and business of the society."

Now, it is generally I fear a much pleasanter task to dilate upon what one is going to do than to hold up for discussion what has actually been done. Yet we can look back upon 1908 as a year which has at least been full of subject matter for consideration: We have not been idle; much has been given, and much received. We have heard and have debated more or less fully some 60 papers, the majority of which have undoubtedly borne the impress of considerable thought and labor, and some of which deserve high commendation as original contributions to the progress of medicine. A pleasant and generous spirit appears to have pervaded the discussions, and I think they have been generally enjoyed.

Our present plan of making an annual programme appears to work well, and I cannot make any suggestions for its improvement. I know that there has been here and there a feeling that sometimes our evenings have been rather full. That may now and then have been the case, and perhaps the affairs of the various committees may for that reason have been thrown a little in the background. On the other hand, I believe that to have been an error in the right direction, because unfortunately we cannot rely upon every member who puts his name down for a paper, being present to read it, and once or twice we have been rather shabbily provided for. On the whole I am in favor of beginning our evenings early and having one table well supplied.

Besides the reading of papers, we have had as a prominent part of our programme the exhibition of cases, specimens, and apparatus, and our thanks are specially due to Dr. Armstrong, and yet more to the gentlemen whose patients have appeared before us, for the trouble they have taken to make this an interesting feature of our meetings. It has, however, in spite of all efforts, not yet attained to the place of importance which it ought to hold among us. It will lie with the new president and his associates to decide what is the best plan whereby they may more deeply interest members in bringing cases before us. This might be made, perhaps, the most valuable department of our meeting; but it needs the hearty and active co-operation of those who are seeing out-door patients at the various clinics of the city. Some of us also are handicapped in so far that in order to see the lesions with which we have to deal, special apparatus is required, and such apparatus the society does not possess. We need such a combination of darkness and light as is suitable to the deeds we do. These instruments we must of course have. They will cost only a few dollars, perhaps $10, $15 or $20 and I now for my part offer to subscribe $5.00 of that amount. I understand that the roo min which we now are is about to be handed over to the society for its individual use. There can, therefore, be no objection to our putting in what apparatus is required. As the idea developes possibly it may be deemed advisable to add an X-Ray, and other more expensive apparatus for purposes of demonstration.

I should like to enquire at this juncture what becomes of those portions of the human form divine, which are being daily plucked from our fellow citizens. Doubtless they are scattered far and wide throughout this part of the country. They belong to all sorts and conditions of men, from the appendix of the society belle down or up to the biceps of "the horny-handed sons of toil," and these specimens are dead. Death levels all men; why not bring them together in the flesh, as they are already in the spirit? The colleges do not want them all; many of our number have no access to either college; we might have a useful museum here through contributions from men throughout the state. Perhaps our next president may see fit to bring such an idea to fruition.

There is a matter which has long appeared to me to be of vital importance, on which I have laid stress here, on several occasions, and in connection with which two committees were appointed last January: I refer to the indifferent part which is played by the medical profession in the public life of the family, the city, the state, and the nation. With your approval, forcibly expressed in the case of certain of our members a committee was appointed to provide at intervals, suitable papers for the journals of the city, with the object of educating the public on subjects which it is necessary for the general welfare that they should understand. I saw personally two editors, and each undertook to publish such contributions, one of them being indeed almost enthusiastic on the subject, while the other dwelt upon the necessity of the paper being written in a short, crisp and attractive form. A number of contributions were sent to the former, but with the exception of one which was worked over and re-written, by one of our number, they were merely excerpts from medical journals, and not one of them has been published. This may appear to be failure; but the matter is far too important to be allowed to die down. Our methods must be altered. It is obvious that the editors want something very short, and specially written for the occasion, and perhaps it is too much to expect a society of the size of ours to produce a constant relay, once a week, or even once a month, of papers which it will pay the daily journals to print. Our refuge, therefore, lies in co-operation. As a matter of fact the central authorities of the American Medical Association should undertake the work, and I should be glad if tonight this society would pass a resolution to that effect to be forwarded to these authorities. But as they may never act, would not it be well for us to get in touch with the societies of the larger cities of Georgia and have a programme written for the year of unsigned papers to be produced by able writers, each to be published in all the newspapers of Georgia which desire them? I look upon this as a matter of vital consequence, and shall be glad to hear it discussed. The other committee to which I have referred had a similar object. Unfortunately that member who kindly undertook to guide it, was so long prostrate with typhoid fever that nothing has been done towards initiating that series of public lectures which we hoped to see in full blast this winter. I notice that a lady has come forward, and has already started something of the kind in this building, though not on medical lines. Possibly her forces and ours might be united, and accomplish something.

It is high time that scientific men, rather than the charletans of the advertising columns of the public prints should guide medical opinion among the laity. We never have done, and we are not now doing a tithe of our sacred duty. Our talents are buried in a napkin; our light is hid under a bushel. Something, indeed, a great deal, is being done to kill out tuberculosis, and our society has helped a little this year; but venereal diseases, infectious fevers, and rabies also require our strenuous attention. Publicity is specially needed in the matter of venereal diseases in order that women may have fair play in the game of matrimony.

We have an excellent state board of preventive medicine, of whose secretary we have special reason to be proud, but would not their hands be greatly strengthened by our moral support? Why do we have hundreds of cases of rabies being treated each year in Atlanta? And more than 40 at the present moment? Is it simply because of a defective insight, and energy among the authorities at Washington? An element of weakness in the practice of state control of legislation is seen here. The whole country should muzzle its dogs for a year and quarantine any which enter it from without. In Britain rabies has in this manner being stamped out. Germany, I believe has done as much for its dogs and its citizens, which proves that a long land frontier it not an insurmountable barrier to success. If the national authorities cannot, or will not act, why should not Georgia make the attempt? Such would be at least an object lesson to others, and would show evidence that this state is farseeing and progressive in its ideas as well as grand in its imperial title.

Another matter which we have attempted to improve, is that of the relationship of doctor and patient in regard to collections. It is a mere truism to say that a debt is never a bond of union, but always tends rather to separation, while it is only reasonable as well as a universal experience that "short debts make long friends." There can be no doubt that the medical profession on the whole, is shamefully abused in this respect, and that an improvement would be a benefit to the community at large. It is interesting to observe that our idea of a central collector has recently been urged by Dr. MacCormack, the well-known organizer of much that is good in the American Medical Association, He also is strongly advising a greater uniformity of fees, a question to consider which one of our committees was appointed.

But I have said enough; and it remains only to thank you for your past kindly oversight of my defects as your presiding officer, and more specially for having honored me so highly as to have elected me three times to that place of importance. I have enjoyed every meeting, and I have learned a great deal from your transactions.

I had hoped to have here tonight a new gavel, which I was going to ask the society to accept as a small remembrance, but Atlanta is not yet big enough to keep many of these in stock, and if you will allow me, I shall hand it over to the new president at his inauguration in January. I again thank you, and wish you the old wish—A merry Christmas and happy New Year.

